# Roles of transducin-like enhancer of split (TLE) family proteins in tumorigenesis and immune regulation

**DOI:** 10.3389/fcell.2022.1010639

**Published:** 2022-11-11

**Authors:** Guiping Yu, Yiqi Chen, Yuwen Hu, Yan Zhou, Xiaoling Ding, Xiaorong Zhou

**Affiliations:** ^1^ Department of Immunology, Nantong University, School of Medicine, Nantong, China; ^2^ Department of Cardiothoracic Surgery, The Affiliated Jiangyin Hospital of Nantong University, Jiangyin, China; ^3^ Department of Periodontology, The Affiliated Nantong Stomatological Hospital of Nantong University, Nantong, China; ^4^ Department of Gastroenterology, The Affiliated Hospital of Nantong University, Nantong, China

**Keywords:** TLE family proteins, transcriptional repressors, tumorigenesis, inflammation, cancer therapy

## Abstract

Mammalian transducin-like enhancer of split family proteins (TLEs) are homologous to *Drosophila* Groucho (Gro) and are essential transcriptional repressors. Seven TLE family members, TLE1-7, have been identified to date. These proteins do not bind DNA directly; instead, they bind a set of transcription factors and thereby inhibit target gene expression. Loss of TLEs in mice usually leads to defective early development; however, TLE functions in developmentally mature cells are unclear. Recent studies have revealed that TLEs are dysregulated in certain human cancer types and may function as oncogenes or tumor suppressors in different contexts. TLE levels also affect the efficacy of cancer treatments and the development of drug resistance. In addition, TLEs play critical roles in the development and function of immune cells, including macrophages and lymphocytes. In this review, we provide updates on the expression, function, and mechanism of TLEs; discuss the roles played by TLEs in tumorigenesis and the inflammatory response; and elaborate on several TLE-associated signaling pathways, including the Notch, Wnt, and MAPK pathways. Finally, we discuss potential strategies for targeting TLEs in cancer therapy.

## Introduction

Transcriptional corepressors, including CtBP, NCoR, SMRT, and TLE family proteins (TLEs), constitute a class of proteins with gene expression inhibitory functions (Cinnamon and Paroush, 2008; Mottis et al., 2013; Ramakrishnan et al., 2018; Chen, 2021). Mammalian TLEs are homologs to the *Drosophila* corepressor Groucho (Gro), which is the only Gro/TLE family member and plays a crucial role in fly development (Jennings and Ish-Horowicz, 2008). In contrast to transcription factors (TFs), TLEs do not bind directly to DNA but are recruited by TFs to inhibit the expression of target genes (Grivas and Papavassiliou, 2013; Adams et al., 2018). Studies in *Drosophila* have found that Rdp3, a homolog of mammalian histone deacetylase 1 (HDAC1), binds to the Gro protein and inhibits target gene expression through histone deacetylation (Mannervik and Levine, 1999). Consistently, mammalian TLEs are reported to recruit different HDACs and suppress transcription in different cell contexts. In addition, recent studies showed that Gro/TLE proteins were mostly locally enriched at gene transcriptional start sites, where H3 and H4 histones were hypomethylated, and that Gro/TLEs may suppress gene expression through an RNA-pausing mechanism (Kaul et al., 2014). These data suggest that various epigenetic mechanisms may contribute to TLE-mediated transcriptional suppression.

Numerous studies have demonstrated that Gro/TLEs are critical for animal development and tissue homeostasis (Chen and Courey, 2000; Cinnamon and Paroush, 2008; Turki-Judeh and Courey, 2012a; Agarwal et al., 2015). Further evidence from recent studies showed that genetic deletion of TLE genes in mice could cause significant defects, especially in the hematopoietic, neuronal, and musculoskeletal systems (Gasperowicz et al., 2013; Wheat et al., 2014; Ramasamy et al., 2016). Moreover, TLEs are abnormally expressed in various tumors and are involved in tumorigenesis (Dayyani et al., 2008; Holmes et al., 2012; Okada et al., 2017; Ring et al., 2018; Palit et al., 2019; Wang et al., 2020). TLEs also regulate the development of progenitor immune cells, and their function in mature immune cells is increasingly being recognized (Wheat et al., 2014; Ramasamy et al., 2016; Xing et al., 2018; Zhang et al., 2019). Disrupted cell differentiation and immune suppression are hallmarks of cancer; therefore, it is speculated that abnormal TLE levels may impact not only tumor cells but also the tumor microenvironment. In this review, we first introduce recent progress in understanding the function, expression, and regulation of TLEs; then, we elaborate on the role played by TLEs in tumorigenesis and immune cells; and finally, we offer perspectives on targeting TLEs for cancer therapy.

## Overview of TLE protein structure, expression, and function

**FIGURE 1:**
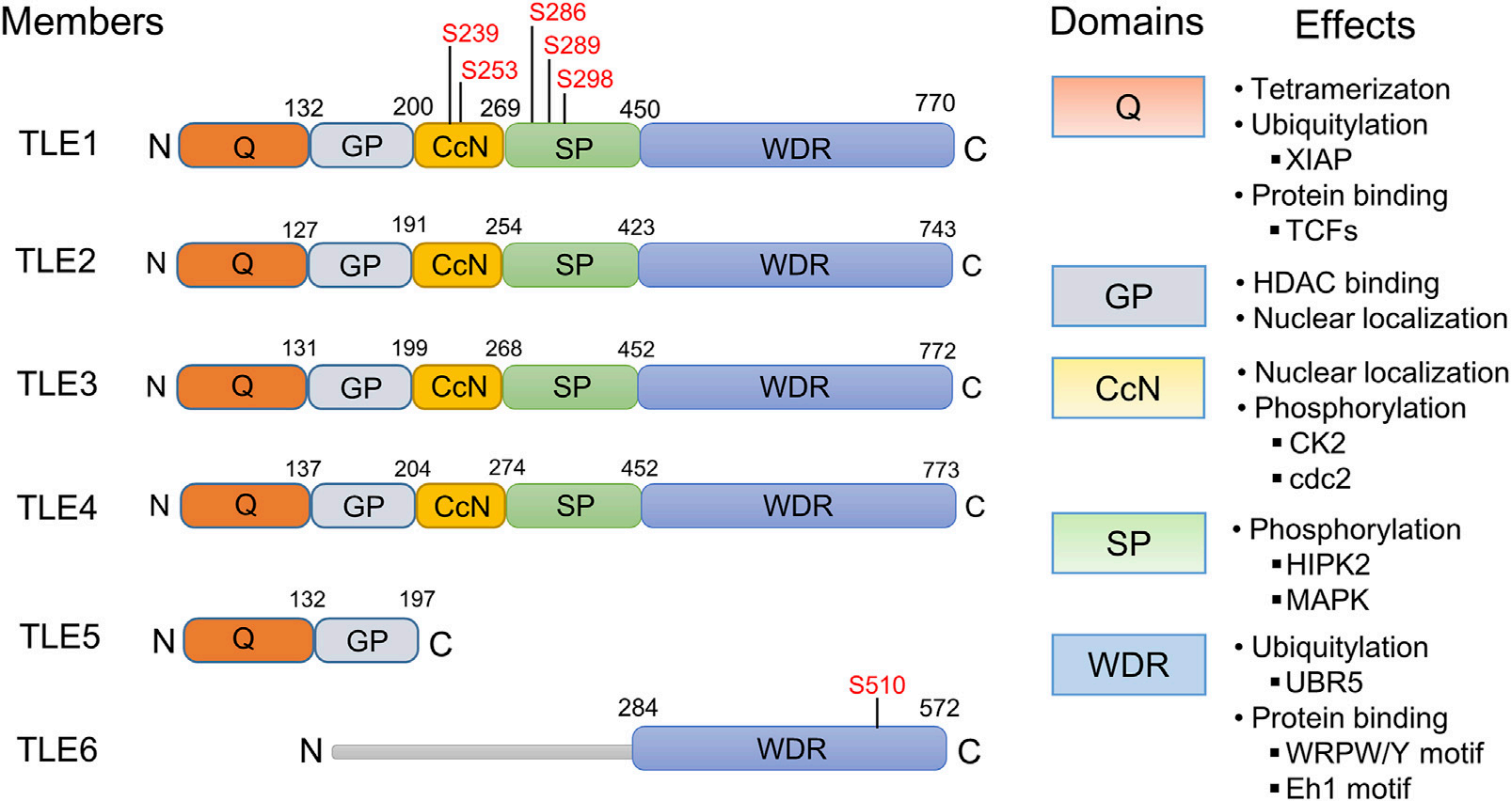
Protein structures, domains, and functions of human TLE family members. The structure of human TLE family members TLE1-6 is displayed. The phosphorylation sites in the Gly-Pro-enriched (GP) and CcN domains are labeled. Domain-specific effects, not necessarily determined from studies of human TLE molecules, are presented. Protein‒protein binding between TLEs and TCF/LEF1 family proteins (TCFs) is mediated by the TLE Q domain (Levanon et al., 1998; Chodaparambil et al., 2014; Flack et al., 2017), while proteins containing the WRPW/Y motif (Jennings et al., 2006; Mahadeveraju et al., 2020) and Eh1 motif (Milili et al., 2002; Goldstein et al., 2005) bind to the TLE WDR domain. The WDR domain of TLEs interacts with the E3 ubiquitin ligase UBR5 (Flack et al., 2017), and the stability of TLEs is regulated by UBR5-mediated ubiquitination and subsequent protein degradation in proteasomes. Interaction between the GP domain and histone deacetylases (HDACs) is thought to contribute at least partially to TLE-mediated transcriptional suppression (Yu et al., 2001), and GP is required for nuclear localization of TLEs (Turki-Judeh and Courey, 2012b); however, the GP domain in human TLE5 differs from that in long-type TLEs and cannot recruit HDACs (Tetsuka et al., 2000; Yu et al., 2001). Phosphorylation of the CcN and SP domains by CK2 (Nuthall et al., 2004), cdc2 (Nuthall et al., 2002), HIPK2 (Choi et al., 2005; Ciarapica et al., 2014), or MAPK (Hasson et al., 2005; Hasson and Paroush, 2006) regulates the suppressive activity of TLEs. TLE6 does not include any identifiable Q, GP, CcN, or Ser-Pro-enriched (SP) domains. Within the WDR domain of TLE6, a PKA-dependent phosphorylation site (S510) is conserved among species and plays a critical role in TLE6 function (Alazami et al., 2015).

The mammalian TLE family comprises seven family members, TLE1-7. TLE7 seems to be expressed at a minimal level, and although its function has not been reported, it is included in this review. As shown in Figure 1, TLE1-4 are long TLE members, featuring a glutamine-enriched N-terminal known as the Q domain and a C-terminal domain that includes WD repeats (WDRs) (Chen and Courey, 2000; Li, 2000). The WDR domain is the most highly evolutionarily conserved domain, with 80% identity among TLE members, and is critical for the interaction between TLEs and many TFs (Chen and Courey, 2000; Li, 2000; Milili et al., 2002; Goldstein et al., 2005; Jennings et al., 2006; Mahadeveraju et al., 2020). The Q domain is required for the tetramerization of TLE proteins, which is essential for TLE-mediated transcriptional suppression (Song et al., 2004; Chodaparambil et al., 2014). The less conserved central region contains three domains, the Gly-Pro-enriched (GP), CcN, and Ser-Pro-enriched (SP) domains, and posttranslational modification of these domains, such as phosphorylation, is involved in the regulation of TLE suppressive activity (Nuthall et al., 2002; Nuthall et al., 2004; Hasson et al., 2005; Turki-Judeh and Courey, 2012b; Kwong et al., 2015). The CcN domain includes a conserved nuclear localization signal (NLS), and the phosphorylation of this domain may dictate the subcellular localization of TLEs (Stifani et al., 1992). Other domain-specific effects are shown in Figure 1.

TLE5 and TLE6 are short-type TLE family proteins. TLE5, also known as AES, lacks the conserved WDR domain and includes only a Q- and C-terminal domain, which are structurally similar to the GP domain in long-type TLEs. TLE5 is thought to be the dominant negative isoform of long TLEs and likely functions by binding to the Q domain of long-type TLE proteins, thereby inhibiting formation of the TLE homotetramer required for transcriptional suppression (Wang et al., 2002; Wang et al., 2004). However, many studies have suggested that TLE5 is a multifunctional protein that can positively or negatively modulate the transcriptional activities of many TFs (Tetsuka et al., 2000; Yu et al., 2001; Swingler et al., 2004; Zhang et al., 2008; Beagle and Johnson, 2010; Chanoumidou et al., 2018). TLE6 is highly expressed in the reproductive system, and its mutation is linked to female infertility (Li et al., 2008; Yu et al., 2014; Lin et al., 2020; Mao et al., 2021; Zhang et al., 2021). It possesses a conserved WDR domain and a highly divergent N-terminal region. However, TLE6 lacks Q, GP, CcN, and SP domains and thus exhibits the lowest identity with other TLE family members (Feng et al., 2020).

TLE family members are widely expressed in many tissues, but the expression profiles of individual TLE proteins have not been extensively examined. Some studies have shown that long-type TLE members exhibit an overlapping but unique expression profile (Hoffman et al., 2008; Chytoudis-Peroudis et al., 2018). For example, Liu et al. found that TLE1, TLE2, and TLE3 are coexpressed in some epithelial tissues and that their levels are relatively high in developmentally immature proliferating epithelial cells but diminished in terminally differentiated cells, whereas in cervical squamous metaplasia and carcinomas, the expression levels of these TLE members are elevated (Liu et al., 1996). In addition, TLE members were found to be expressed in a complementary pattern in developing mouse neurons and the pancreas (Yao et al., 1998; Theis et al., 2021). These results suggest that TLE expression is spatiotemporally regulated and that different TLEs may exhibit specific activity in different cell contexts.

Phenotypic analysis of mice with genetic deletion of individual TLE members revealed some previously unappreciated functions of TLEs. For example, TLE1 gene-knockout (Tle1^−/−^) mice exhibited growth retardation, with apparent defects in the lung, intestine, and skin development (Ramasamy et al., 2016). In addition, the expression of inflammatory cytokines was increased in multiple organs in Tle1^−/−^ mice, leading to an unrestrained systemic inflammatory response, suggesting that TLE1 is a critical regulator of development and inflammation (Ramasamy et al., 2016). TLE2 is reported to play a role in the developing mouse pancreas, but surprisingly, mice with global deletion of the Tle2 gene showed no discernable phenotype (Roth et al., 2010; Theis et al., 2021). Global deficiency of Tle3 caused embryonic death, which was associated with abnormal placental development (Gasperowicz et al., 2013), and tissue-specific genetic depletion demonstrated that TLE3 is required for various biological processes, including bone development (Kokabu et al., 2013), myogenic differentiation (Kokabu et al., 2017), lipid storage and thermogenesis (Pearson et al., 2019).

Wheat et al. reported that Tle4^−/−^ mice were developmentally runted and died from hematopoietic and skeletal defects at approximately 4 weeks of age (Wheat et al., 2014). Interestingly, Zhang et al. independently established Tle4^−/−^ mice and found that some of these mice died early, but the surviving Tle4^−/−^ mice showed no observable abnormalities compared with Tle4^+/+^ littermates (Zhang et al., 2019). The reason for this discrepancy is unclear. Nevertheless, both groups observed significant defects in hematopoiesis and bone development in the early development of Tle4^−/−^ mice (Wheat et al., 2014; Zhang et al., 2019; Shin et al., 2021a). Tle5^−/−^ mice showed initial growth retardation due to defective bone development but quickly established a growth pattern; the phenotype was milder than that of Tle4^−/−^ mice (Wang et al., 2002; Wang et al., 2004). Allen et al. established transgenic mice expressing Tle1, Tle5, or both and found that Tle1 transgenic mice spontaneously developed lung cancer, whereas Tle5 transgenic mice were normal (Allen et al., 2006), suggesting that TLE1 might be a lung-specific oncogene and that its tumor-promoting activity might be antagonized by TLE5 (Allen et al., 2006).

The phenotype of individual TLE gene-knockout mice suggests that different TLE family members likely play unique roles despite a certain level of redundancy. The possible reasons for the differences are as follows: 1) Individual TLE members possess unique features. Through a common region in the WDR domain, TLEs bind many TFs containing WPRW/Y and Eh1 motifs (Jennings et al., 2006), and some of these TFs may express atypical WPRW/Y and Eh1 motifs. Therefore, different TLEs may preferentially bind to different TFs, resulting in distinct biological effects that cannot be easily determined by overexpressing individual TLE molecules *in vitro* (Buscarlet and Stifani, 2007). 2) Both individual TLE members and their interacting TFs are differentially expressed. 3) TLE activity is regulated by a variety of posttranslational modifications, such as phosphorylation (Choi et al., 2005; Ciarapica et al., 2014), ubiquitination (Nuthall et al., 2004; Flack et al., 2017), and SUMOylation (Ahn et al., 2009; Lee et al., 2012). In the future, conditional knockout mice will be useful for analyzing the role played by TLE members in different tissue and cell types. In addition, many new technologies, including single-cell mRNA sequencing, spatial transcriptome analyses, and mass spectrometry analyses, may help deepen our understanding of the regulation and function of TLE members.

## TLEs in tumorigenesis and cancer therapy

### Tumor suppressive activity of TLEs

Studies have shown that TLE family proteins are abnormally expressed in certain cancer types. For example, TLE1 is highly expressed in synovial sarcoma and has been used as a sensitive and specific diagnostic marker (El Beaino et al., 2020). A series of studies have identified TLEs as tumor suppressors in blood cancers. For instance, the AML1-ETO fusion protein is a critical oncogenic driver of acute myeloid leukemia (AML), but this fusion protein alone cannot transform normal cells into malignant cells, suggesting that other factors are required for tumorigenesis (Mulloy et al., 2002). Dayyanid et al. found that loss of TLE1 and TLE4 cooperated with AML1-ETO fusion protein expression to induce tumorigenesis (Dayyani et al., 2008). In addition, overexpression of TLE1 or TLE4 inhibited the *in vitro* proliferation of lymphoma cells harboring AML1-ETO, and knocking down a TLE homolog promoted the growth of lymphoma in a zebrafish model (Dayyani et al., 2008). Shin et al. also found that TLE4 deficiency and AML1-ETO expression synergistically induced AML (Shin et al., 2016) and that loss of TLE4 induced resistance to chemotherapy in AML cell lines by activating the Wnt signaling pathway (Shin et al., 2016). Fraga et al. found that the CpG island in the TLE1 gene promoter is frequently hypermethylated, causing epigenetic inactivation of TLE1 in various blood tumor cells, including AML, chronic myeloid leukemia (CML), and non-Hodgkin’s lymphoma (Fraga et al., 2008). In T-cell acute lymphoblastic leukemia (T-ALL), the expression of TLE1 is significantly decreased, which is associated with disease recurrence and a poor prognosis (Brassesco et al., 2018; Aref et al., 2021).

In pancreatic cancer, high levels of TLE1 are associated with a better prognosis, and overexpression of TLE1 inhibits tumor cell proliferation and migration (Wang et al., 2020). In addition, high TLE1 expression is common in gastric cancer and is related to a better prognosis. In liver cancer, the protumorigenic effect of microRNA (miRNA)-657 was found to be related to TLE1 downregulation, suggesting that TLE1 may act as a tumor suppressor in liver cancer (Zhang et al., 2013). In addition, upregulation of TLE2 expression is associated with a favorable prognosis in pancreatic and bladder cancer (Wu et al., 2019; Hu et al., 2020). In triple-negative breast cancer, the presence of TLE3 is associated with a better prognosis (Kashiwagi et al., 2017). In colorectal cancer, high RNF6 expression promotes tumor cell proliferation and metastasis, and the effect is related to RNF6-mediated TLE3 ubiquitination and degradation, followed by increased Wnt pathway activation (Liu et al., 2018). In addition, TLE5 inhibits the metastasis of colorectal cancer and prostate cancer by inhibiting the Notch and androgen receptor (AR) signaling pathways (Sonoshita et al., 2011; Okada et al., 2017; Wang et al., 2021a). Together, these results suggest that TLE proteins may function as tumor suppressors in various blood and solid tumors.

### Tumor-promoting activity of TLEs

**FIGURE 2:**
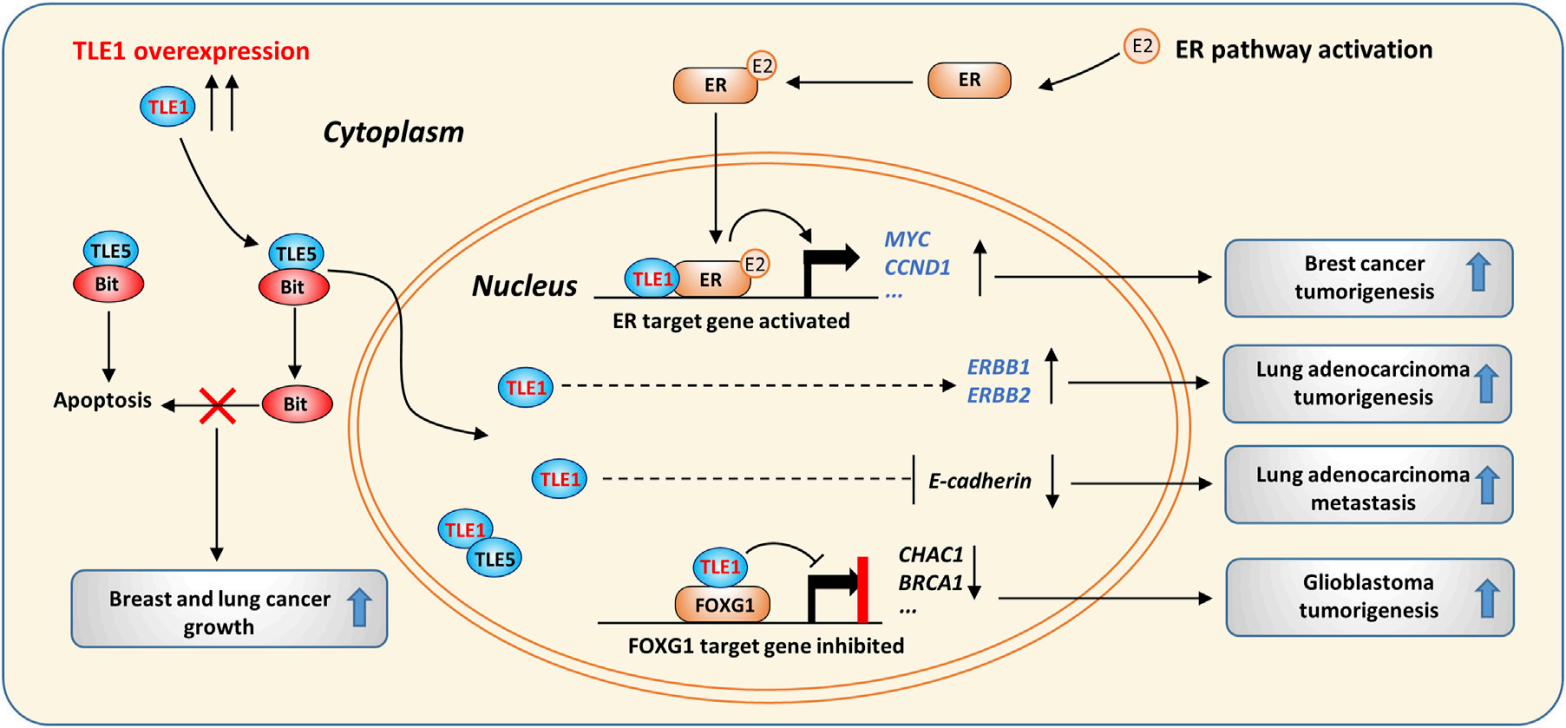
The oncogenic activity of TLE1 in cancer. TLE1 suppresses cytosolic Bit/TLE5-induced apoptosis by forming a TLE1/TLE5 complex, which is mainly localized in the nucleus. The ER pathway is activated in the presence of estrogen (E2), and pathway activation promotes breast cancer growth. TLE1 forms a complex with activated ER and enhances ER target gene expression. TLE1 overexpression increases the expression of ERBB1/2 and induces EMT, which was found to contribute to lung adenocarcinoma tumorigenesis in transgenic mice. In GBM, the TLE1/FOXG1 complex suppresses a cluster of FOXG1 target genes, thereby promoting GMB tumorigenesis by activating the Notch signaling pathway.

TLE1 may function as an oncogene in certain tumors (Figure 2). TLE1 transgenic mice were reported to spontaneously develop lung cancer, likely due to enhanced ERBB1 and ERBB2 expression (Allen et al., 2006). High levels of TLE1 in lung adenocarcinomas are associated with a poor prognosis, suggesting that TLE1 may be a lung tissue-specific oncogene (Allen et al., 2006; Ma et al., 2021). In addition, overexpression of TLE1 suppressed the expression of E-cadherin, induced epithelial-mesenchymal transition (EMT) in human lung cancer cells, and promoted the growth and metastasis of transplanted tumors in mice (Yao et al., 2014a; Yao et al., 2016).

Analysis of clinical samples demonstrated that TLE1 levels are significantly higher in invasive breast cancer than in noninvasive breast cancer (Brunquell et al., 2012; Lee et al., 2017). TLE1 promotes estrogen receptor (ER) target gene expression in breast cancer, leading to increased tumor cell proliferation (Holmes et al., 2012). Mechanistically, TLE1 directly binds the histone tail of local nucleosomes and facilitates the binding of ER to DNA, thereby enhancing the transcription of a cluster of ER target genes, including MYC and CCND1 (Figure 2). TLE1 was shown to interact with estrogen receptor related-γ (ERR-γ) and function as a coactivator in a kidney cell line (Hentschke and Borgmeyer, 2003). Interestingly, TLE3 was found to interact with PPARγ to promote PPARγ-dependent gene expression during white adipose cell differentiation (Villanueva et al., 2011). Therefore, TLEs can act as transcriptional coregulators and positively or negatively control target gene expression in different cell contexts.

In addition, TLE1 overexpression was reported to induce apoptosis resistance and promote anchorage-independent growth in breast and lung cancer cells (Brunquell et al., 2012; Yao et al., 2016). When normal cells lose integrin-mediated cell attachment, Bit, a mitochondrial protein, is released into the cytosol and induces anoikis by forming a complex with TLE5 (Jan et al., 2004). However, in cancer cells, overexpression of TLE1 can suppress anoikis by competing with TLE5 for Bit binding and sequestering TLE5 in the nucleus (Brunquell et al., 2012; Yao et al., 2014b) (Figure 2). EMT and anoikis resistance are known to promote the migration and survival of cancer cells that lose their cell–extracellular matrix (ECM) interactions (Cao et al., 2016), and these data suggest that TLE1 overexpression may promote lung and breast cancer metastasis by enhancing cancer cell migration and survival.

Meningioma is a common tumor of the central nervous system. TLE2 and TLE3 expression is upregulated in high-grade meningioma (Cuevas et al., 2005). TLE3 expression is also increased during melanoma progression, and overexpression of TLE3 promotes melanoma cell proliferation and tumor progression (Ogawa et al., 2019). Notably, the protumorigenic activity of TLE3 in melanoma can be blocked by treatment with HDAC inhibitors. Given that TLE3/HDACs can inhibit Wnt/β-catenin signaling and that Wnt activation has adverse effects on the growth of melanoma cells, these results suggest that TLE3 might promote melanoma progression by regulating Wnt signaling (Ogawa et al., 2019).

In glioblastoma (GMB), the expression of TLE1 is upregulated and associated with a poor prognosis; further studies revealed that TLE1 forms a complex with FOXG1 to inhibit the expression of CHAC1, a negative regulator of the Notch signaling pathway, resulting in overactivation of the Notch signaling pathway and accelerated GMB progression (Figure 2) (Verginelli et al., 2013; Dali et al., 2018). TLEs are highly expressed in cervical and colonic carcinoma cells compared to normal epithelial cells, and the increase in TLE expression is correlated with activation of the Notch signaling pathway (Liu et al., 1996). Constitutive activation of Notch signaling is protumorigenic in certain cancer types, such as T-ALL (Dayyani et al., 2008; Sanchez-Martin and Ferrando, 2017), mammary and salivary gland tumors (Boyle et al., 2017; Xie et al., 2020), and lung adenocarcinomas (Allen et al., 2011; Wael et al., 2014), but whether TLEs promote the progression of these tumors by regulating the Notch pathway has not been thoroughly examined.

### TLEs are involved in resistance to cancer therapy

The TLE level affects cancer cell responses to anticancer therapy. In a prostate cancer study, TLE3 deficiency was linked to resistance to AR inhibitors (Palit et al., 2019). In pancreatic cancer cells, increased TLE2 expression induced cell cycle delay in the S-phase and sensitivity to gemcitabine (Hu et al., 2020). In AML cell lines, loss of TLE4 and Wnt signaling pathway activation increased the expression of proinflammatory genes and induced resistance to chemotherapy (Shin et al., 2016). In studies of ovarian and breast cancers, increased TLE3 expression was related to a higher response rate to taxane-based chemotherapy, but TLE3 expression showed no predictive value in treating breast cancer at an early stage (Samimi et al., 2012; Ring et al., 2018). The level of TLE3 expression was also found to be positively correlated with the sensitivity of breast cancer cells to tamoxifen treatment (van Agthoven et al., 2009). TLE3 binds FOXA1 to inhibit the expression of ER target genes in breast cancer cells, which may explain the influence of TLE3 on the sensitivity of tumor cells to tamoxifen treatment (Jangal et al., 2014). In addition, Kornspan et al. found that overexpression of TLE3 but not TLE1 enhanced non-small cell lung cancer (NSCLC) cell line sensitivity to taxane treatment (Kornspan et al., 2021). Together, these results suggest that altered TLE expression may impact the sensitivity of certain types of tumors to chemotherapy or targeted therapy, and the mechanisms warrant further investigation.

## TLEs regulate signaling pathways involved in tumorigenesis and immune regulation

### Notch signaling pathway

**FIGURE 3:**
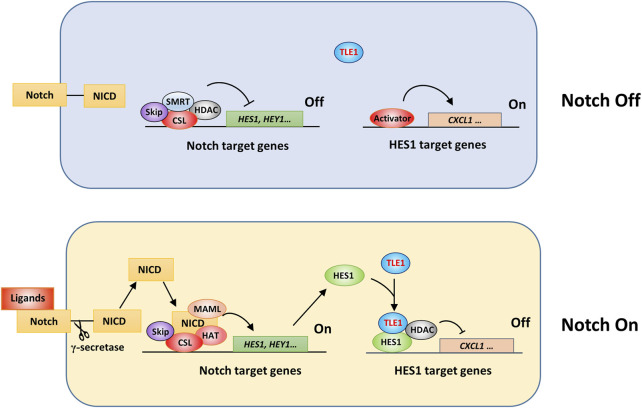
Schematic showing TLE1 regulation of Notch activation effects. In *Drosophila*, corepressor Gro and CtBP proteins are involved in the transcriptional suppression of Notch target genes (Nagel et al., 2005; Guo et al., 2019). In mammalian cells, Notch target gene expression might be inhibited by a suppressive complex comprising CSL, the corepressor SMAT/Skip, and histone deacetylases (HDACs) in the absence of Notch signaling activation (Notch off). Upon Notch activation (Notch On), an activation complex consisting of CSL, NICD, MAML, and histone acetyltransferase (HAT) is assembled to drive the expression of Notch target genes. The interaction of HES1 and TLE1, and likely other TLE members, is critical for the transcriptional suppression of HES1 target genes.

Notch signaling is involved in many physiological processes and diseases, including cancer and immune dysregulation (Ferreira and Aster, 2021). The human Notch system includes five ligands and four membrane receptors (Ferreira and Aster, 2021). Without Notch ligand binding, the corepressor SMRT/SKIP complex is recruited to the promoter of Notch target genes by RBP-J (CSL) and interacts with histone deacetylases (HDACs), thereby suppressing the transcription of Notch target genes (Figure 3) (Contreras-Cornejo et al., 2016). Meanwhile, HES1 target genes, such as the inflammatory factor CXCL1, can be expressed once relevant activators are recruited to the gene promoter (Shang et al., 2016). The binding of transmembrane Notch receptors by ligands on adjacent cell surfaces triggers γ-secretase-dependent cleavage of Notch receptors, leading to release of the Notch intracellular domain (NICD) (Nowell and Radtke, 2017; Zhou et al., 2022). Then, the NICD is translocated into the nucleus and forms a complex with RBP-J and cofactors, including MAML and histone acetyltransferases (HATs), leading to transcriptional activation of Notch target genes, such as HES1 (Espinosa et al., 2010) and HEY1 (Xie et al., 2020). HES1 can form a complex with TLEs and HDACs, thus executing Notch effects by suppressing target gene transcription (Figure 3) (Nowell and Radtke, 2017; Zhou et al., 2022). Therefore, TLEs can regulate Notch effects by triggering suppressive epigenetic modification and inhibiting HES1 target gene expression (Cuevas et al., 2005; Buscarlet et al., 2008; Espinosa et al., 2010; Larabee et al., 2013; Schnell et al., 2015; Shang et al., 2016; Zhang et al., 2019). In addition, TLE5 may recruit HDAC3 to the promoter of Notch target genes in colorectal cancer cells to suppress transcription, and interestingly, these effects depend on unique TLE complexes formed by TLE1 and TLE5 in the nucleus (Sonoshita et al., 2011; Itatani et al., 2016).

### Wnt signaling pathway

**FIGURE 4:**
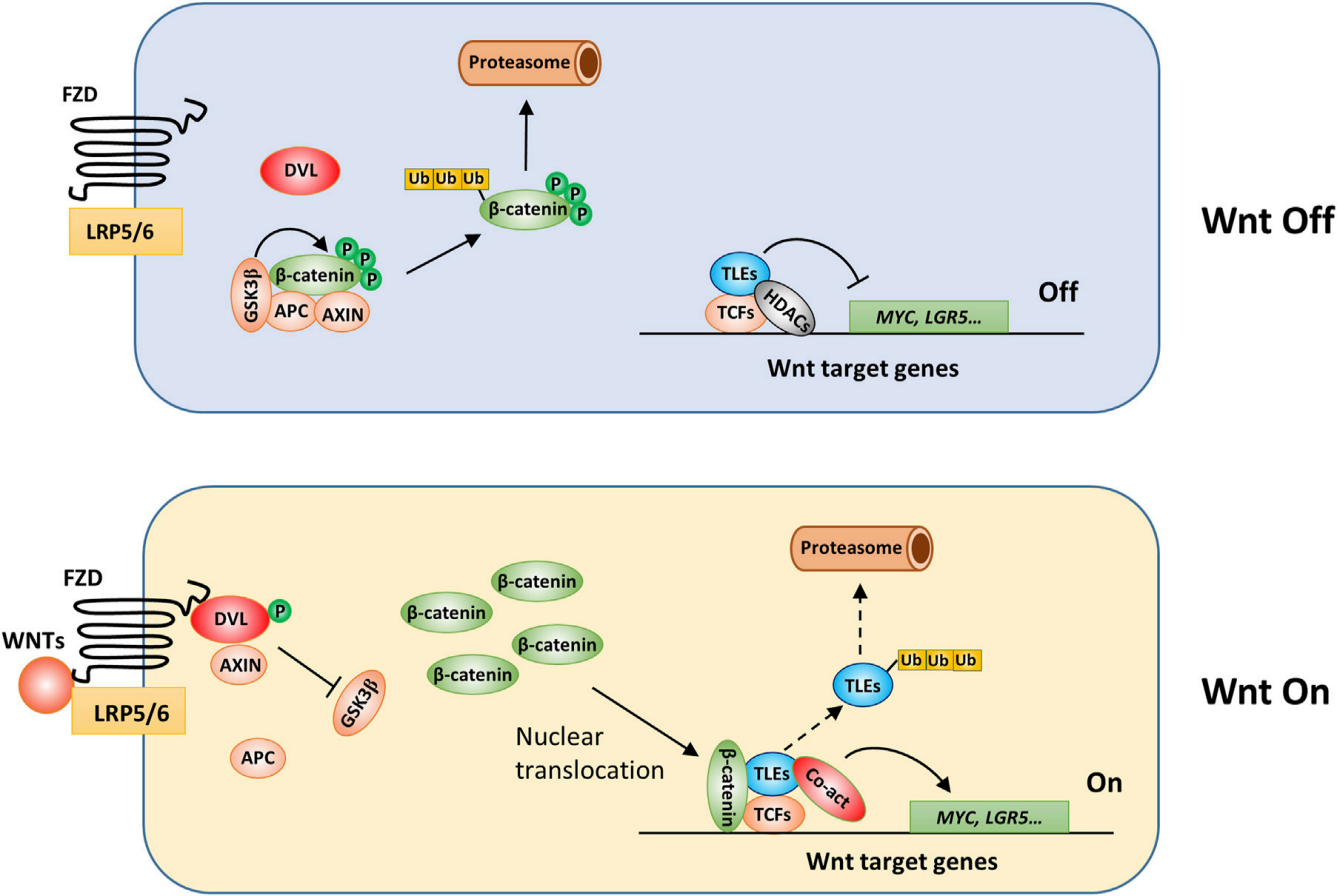
The involvement of TLEs in regulation of classical Wnt signaling. Under baseline conditions, long-type TLEs bind TCF/LEF1 family proteins (TCFs) through their Q domain and HDACs through the GP domain and thus inhibit the expression of Wnt target genes (Brantjes et al., 2001), such as MYC and LRG5 (Bottomly et al., 2010; Kim et al., 2017). TLE5 may interact specifically with TCF3/4 to downregulate Wnt signaling (Costa et al., 2013). Wnt activation induces Dishevelled (DVL) protein phosphorylation and the subsequent dissociation of the *ß*-catenin destruction complex. Therefore, *ß*-catenin accumulates in the cytoplasm. After translocation to the nucleus, *ß*-catenin is recruited by TCFs to the promoter of Wnt target genes, where it forms a complex with coactivators and drives the transcription of Wnt target genes (Duchartre et al., 2016). TLEs may be ubiquitinated and subsequently degraded (Hanson et al., 2012; Flack et al., 2017); however, the precise mechanisms remain unresolved.

Wnt signaling plays a critical role in animal development and participates in many physiological processes, such as stem cell maintenance, wound healing, and immune responses, and its activation leads to protumorigenic effects in many types of malignancies, especially colorectal cancer (Duchartre et al., 2016). Wnt signaling pathways are categorized into classical or *ß*-catenin-dependent and nonclassical or *ß*-catenin-independent pathways (Duchartre et al., 2016). In the classical pathway (Figure 4), Wnt ligands, a group of cysteine-rich glycoproteins, are released into the extracellular matrix and bind to frizzled (FZD) receptors and LRP5/LRP6, triggering downstream intracellular signaling events that inhibit *ß*-catenin destruction complex activation and activate *ß*-catenin-dependent signaling (Duchartre et al., 2016). The *ß*-catenin destruction complex is mainly composed of adenomatosis polyposis coli (APC), glycogen synthase kinase 3 (GSK3), and Axin2, and in the absence of Wnt ligand binding, this complex induces phosphorylation and then ubiquitination of *ß*-catenin, which eventually undergoes degradation through the proteasome (Figure 4). In addition, in the nucleus, TCF/LEF1 family proteins (TCFs) form complexes with TLEs and suppress Wnt target gene transcription (Costa et al., 2013; Pai et al., 2017; Santiago et al., 2017). ChIP-seq results suggested that approximately one-half of the genes occupied by TCFs were also occupied by TLEs in mouse stem cells, suggesting that TLEs play an essential role in regulating Wnt target gene expression (Lien et al., 2014). Upon Wnt activation, the *ß*-catenin destruction complex is dissociated, which allows *ß*-catenin to accumulate in the cytoplasm and be translocated to the nucleus, where it binds TCFs, blocking the inhibitory effect of TLEs and thus triggering the expression of Wnt target genes (Ramakrishnan et al., 2018). Ubiquitination and subsequent proteasome-dependent degradation of TLEs may relieve Wnt signaling inhibition and thus promote tumorigenesis. For example, Flack et al. found that Wnt activation in colon cancer cells led to UBR5-dependent TLE3 ubiquitination, changing the TCF-TLE-β-catenin protein complex from a transcriptional inhibitor to a transcriptional activator, although the exact mechanism involved in this activation switching is unclear (Flack et al., 2017).

### EGFR/MAPK signaling pathway

Studies in *Drosophila* suggested that EGFR and Notch signals antagonize each other for correct cell fate specification and that Gro plays a critical role in this antagonism (Hasson et al., 2005; Hasson and Paroush, 2006). Without EGFR ligand binding, Gro may halt the expression of a group of EGFR signaling target genes (Hasson et al., 2005; Hasson and Paroush, 2006). EGFR ligand binding leads to activation of the MAPK signaling cascade (RAS/RAF/MEK/ERK), culminating in ERK phosphorylation and target gene expression. Gro can be phosphorylated through the MAPK signaling pathway, which attenuates Gro-mediated transcriptional suppression, resulting in a rapid response and syngeneic expression of many EGFR genes (Hasson et al., 2005; Hasson and Paroush, 2006). This corepressor-mediated regulatory mechanism is of great significance to ensure that *Drosophila* cells respond quickly to extracellular stimuli, and the mechanism may be conserved in mammalian cells. EGFR is frequently overexpressed in a variety of tumors (Sigismund et al., 2018), and activating mutations of EGFR, RAS, and RAF are the most significant oncogenic drivers in many malignancies, including lung (Chevallier et al., 2021), colorectal (Cancer Genome Atlas Network, 2012), pancreatic (Qian et al., 2020), and skin (Hodis et al., 2012) cancers. MAPK signaling leads to TLE1 phosphorylation and inhibits TLE1 suppressive activity by translocating it from the nucleus to the cytoplasm in tumor cell lines (Zahavi et al., 2017). However, the results of TLE1 immunohistochemistry (IHC) studies in pancreatic and lung cancer tissue samples showed that TLE1 was mainly distributed in the nucleus, even though constitutive MAPK activation was prominent in these two cancer types (Allen et al., 2006; Wang et al., 2020); therefore, whether MAPK regulates the nuclear localization of TLE1 in certain tumors *in vivo* remains to be verified.

### NF-κB signaling pathways

In most cases, NF-κB signaling promotes tumor progression and is thought to be a critical link between cancer and inflammation (Xia et al., 2014; Taniguchi and Karin, 2018). TLEs may modulate the NF-κB signaling pathway through various mechanisms (Tetsuka et al., 2000; Ghosh et al., 2007; Ramasamy et al., 2016; Chen et al., 2020). For example, TLE1 was reported to interact with NOD2 and thus inhibit NF-κB signaling, and TLE1 gene mutation or reduced TLE1 expression was found to be associated with the pathogenesis of inflammatory bowel disease and hepatic ischemia/reperfusion injury (Nimmo et al., 2011; Chen et al., 2020). In T lymphoma cell lines, TLE5 interacted with the NF-κB subunit p65 (RelA) and inhibited p65-driven gene expression (Tetsuka et al., 2000). TLE1 overexpression prevented the translocation of p65 and thus inhibited NF-κB signaling in macrophages, whereas in the absence of TLE1, increased p65 phosphorylation and inflammatory gene expression were observed (Ramasamy et al., 2016). TLE1 was also reported to inhibit NF-κB signaling by interacting with Sirt1, and the effects were not dependent on the deacetylase activity of Sirt1 (Ghosh et al., 2007).

### ER and AR receptor signaling pathways

**FIGURE 5:**
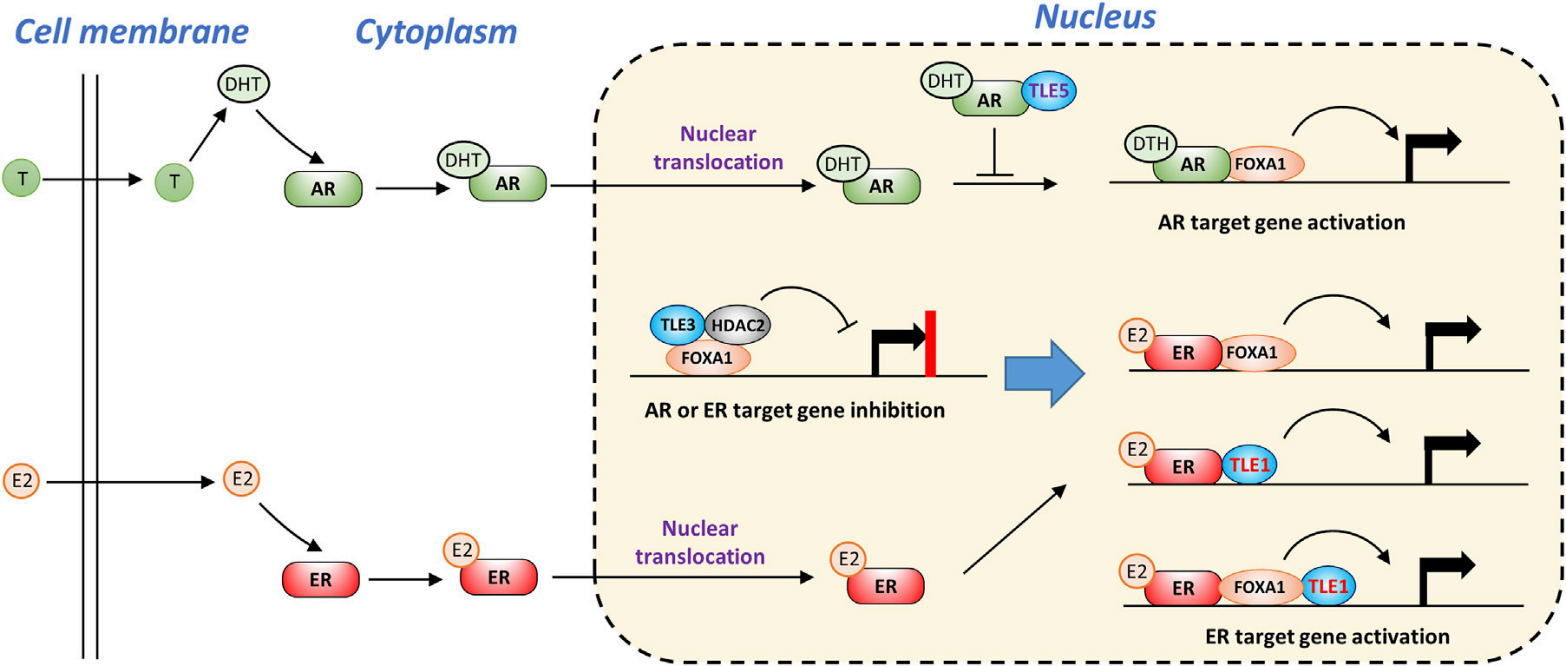
TLEs regulate AR and ER ligand-dependent signaling pathways. Without ligand stimulation, TLE3 is required to prevent inappropriate expression of AR or ER target genes. Upon ligand stimulation, TLE1 functions as a coactivator of ER target gene expression by interacting with ER and FOXA1. TLE5 suppresses AR target gene expression by interfering with AR-DNA interactions.

TLEs are also involved in estrogen receptor (ER) and androgen receptor (AR) signaling pathways (Nakaya et al., 2007; Jangal et al., 2014; Stelloo et al., 2018), and both pathways provide important survival signaling for cancer cells expressing these receptors (Aurilio et al., 2020; Belachew and Sewasew, 2021). As shown in Figure 5, without ligand stimulation, the basal expression of ER or AR target genes is likely suppressed by a complex containing TLE3, FOXA1, and HDAC2 (Jangal et al., 2014; Palit et al., 2019). Upon stimulation with estrogen (E2), ER is activated and translocates into the nucleus. TLE1 may form a complex with ER in the presence or absence of FOXA1, facilitating the binding of ER to a cluster of target genes and leading to increased gene expression (Holmes et al., 2012). Similarly, dihydrotestosterone (DHT), a potent androgen converted from testosterone (T), activates AR and stimulates target gene expression by interacting with FOXA1 (Sahu et al., 2013). TLE5 directly interacts with AR and prevents AR from binding DNA, thus suppressing ligand-dependent AR target gene expression and inhibiting prostate cancer progression and metastasis (Yu et al., 2001; Zhang et al., 2010; Okada et al., 2017) (Figure 5).

## Crosstalk among the TLE-regulated pathways in tumorigenesis

### TLEs regulate cell fate choice and tumor progression

As mentioned earlier, TLEs play vital roles in the development of the lung and pancreas (Metzger et al., 2012; Ramasamy et al., 2016; Theis et al., 2021). High TLE1 expression in lung adenocarcinoma is associated with a poor prognosis (Ma et al., 2021) but, surprisingly, is associated with tumor inhibition and a better prognosis in pancreatic cancer (Wang et al., 2020). These differences may be due to different tumor-initiating cell types and cellular contexts. Similarly, Notch signaling can lead to either protumorigenic or antitumorigenic effects depending on the tissue and cellular context (Nowell and Radtke, 2017; Zhou et al., 2022). Notch signaling may affect tumor development in several ways. For example, Notch activation can induce lateral inhibition in adjacent cells, mediating signals that induce them to adopt a specific cell fate, a mechanism that plays a vital role in multicellular organism development. In lung tissue specifically, Notch activation drives the differentiation of proximal lung progenitor cells toward club cells instead of neuroendocrine (NE) cells (Kiyokawa and Morimoto, 2020; Sharif et al., 2020; Bodas et al., 2021). In the distal lung, Notch activation blocks the AT2 cell transition to AT1 cells (Chen and Liu, 2020; Sharif et al., 2020). AT2 and club cells are the primary origins of lung adenocarcinoma cells (Desai et al., 2014; Behrend et al., 2021), whereas NE cells are the major cell of origin in small-cell lung cancer (Sutherland et al., 2011). Consistently, Notch1 activation led to protumorigenic effects in lung adenocarcinoma and antitumorigenic effects in small-cell lung cancer (Licciulli et al., 2013; Nowell and Radtke, 2017).

Dysregulation of Notch signaling alone may not be sufficient to induce tumorigenesis, but it can promote the generation of pathologically specific cancer types by cooperating with oncogenic drivers, such as KRAS- and EGFR-activating mutations in lung adenocarcinomas or PTEN- and RB-inactivating mutations in small-cell lung cancer (Sriuranpong et al., 2001). As mentioned above, the Tle1 transgene caused spontaneous lung cancer, although no test was performed to determine whether this effect was related to Notch overactivation (Allen et al., 2006). In contrast, Tle1^−/−^ mice exhibited apparent lung hypoplasia, which might be associated with inhibition of Notch-induced lung epithelial cell differentiation (Ramasamy et al., 2016).

The cell fate decision is a critical event in development, and during lineage determination, the cellular proliferation program is likely to be temporarily suspended and later reinitiated for lineage expansion. Thus, Notch activation must be strictly regulated, allowing the fine-tuned control of cell cycle exit and re-entry, which involves a network of feedback mechanisms, including lateral inhibition and lateral induction pathways (Bocci et al., 2020). A handful of Notch direct target genes have been reported, including HES1 (Espinosa et al., 2010), HEY1 (Xie et al., 2020), p21 (Monahan et al., 2009), c-Myc (Weng et al., 2006), and Slug (Niessen et al., 2008), whose dysregulation is frequent in cancer. Notch activation might inhibit cell cycle progression by increasing p21 expression (Noseda et al., 2004; Monahan et al., 2009), but impaired lineage consolidation and additional proliferation-promoting signals from oncogenic events may cooperatively induce the dedifferentiation of mature cells toward proliferating immature cells with cancer stem cell traits (Bi et al., 2016; Venkatesh et al., 2018; Meisel et al., 2020; Sun et al., 2022), thereby driving tumor imitation, metastasis, and drug resistance (Meisel et al., 2020).

**FIGURE 6:**
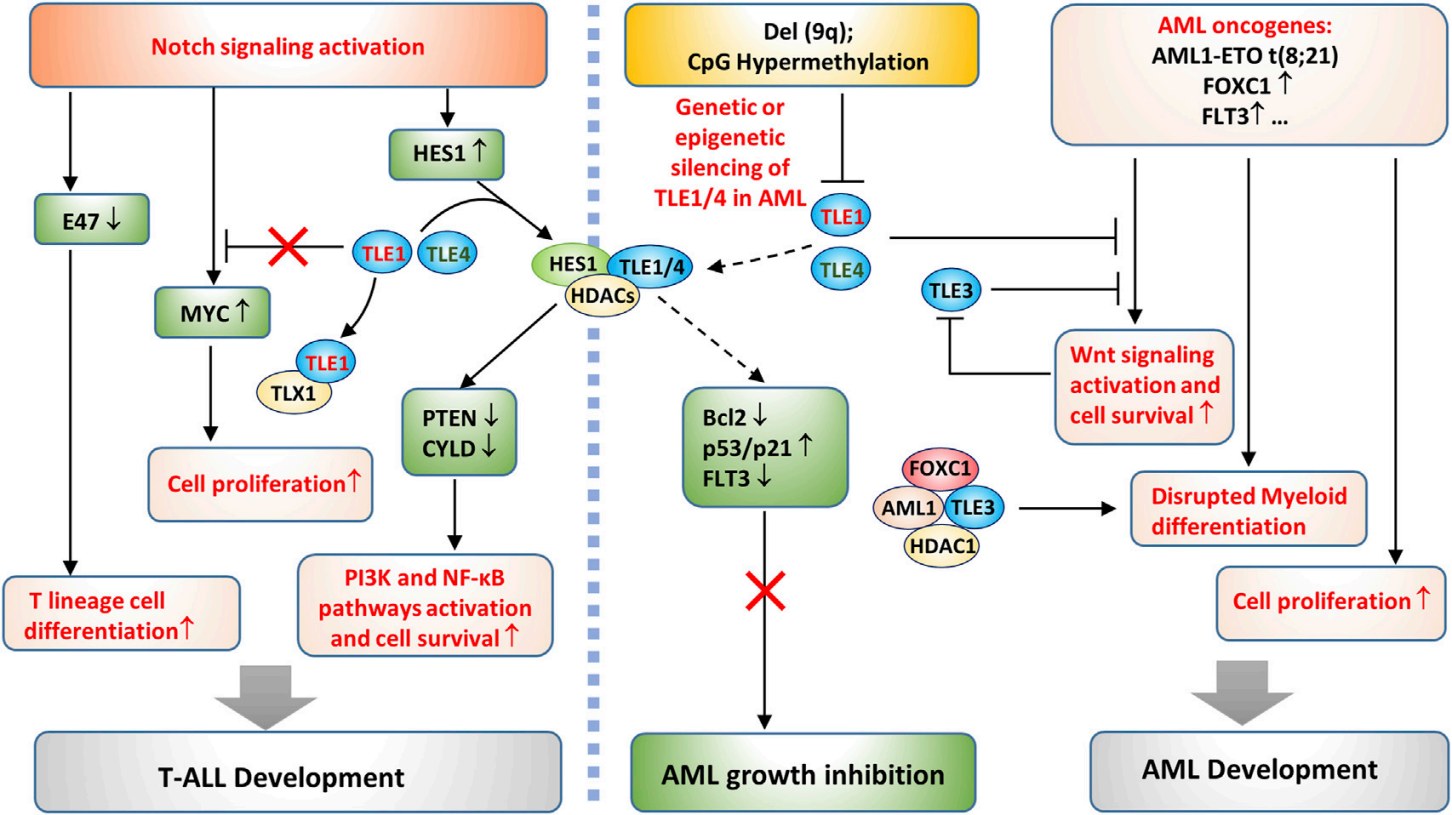
TLE-regulated pathways in AML and T-ALL development. On the one hand**,** deletion of a portion of the long arm of chromosome 9, del (9q), or CpG island hypermethylation in the TLE1 promoter causes genetic or epigenetic silencing of TLE1 and TLE4 in AML cells. The loss of TLE1/TLE4 disrupts the TLE/HES1/HDAC complex, which otherwise inhibits AML growth by increasing p53/p21 and decreasing Bcl-2 expression (Kannan et al., 2013). Loss of HES1/TLEs may also promote AML growth by derepressing FLT3 expression (Kato et al., 2015) and enhancing Wnt signaling activation. On the other hand, AML oncogenic events promote cell proliferation and disrupt the terminal differentiation of myeloid cells, both of which are required for AML development. Notch activation promotes T-ALL development by several means. It directly upregulates the Notch target gene MYC to promote tumor cell survival. Notch activation inhibits E47, which leads to committed T lineage differentiation of T-ALL cells. In addition, Notch activates HES1 expression and suppresses the expression of PTEN and CYLD, thereby enhancing PI3K and NF-κB pathway activation and cell proliferation.

In hematopoiesis, Notch plays a vital role in the determination of cell differentiation into a myeloid or lymphoid lineage (Kondo, 2010). Notch overactivation exerts an oncogenic effect in T-ALL (Koch and Radtke, 2011). In contrast, Notch plays a tumor suppressive role in AML, and reactivating Notch is considered an attractive strategy for AML therapy (Tohda, 2014; Ye et al., 2016). As mentioned above, genetic deletion of TLE1/4 is a synergistic factor in AML development, and the underlying mechanism might be associated with a disrupted lineage choice (Dayyani et al., 2008). In other words, the deletion of TLE1/4 may relieve the Notch-mediated inhibition of myeloid lineage differentiation and thus promote AML tumorigenesis in synergy with oncogenic events, such as AML1-ETO gene translocation, FOXC1 gene amplification, and FLT3 overexpression (Figure 6) (Kishtagari et al., 2020). FOXC1 overexpression is common in AML with wild-type AML1 (Han et al., 2017; Simeoni and Somervaille, 2021), where TLE3 forms a complex with wild-type AML1, FOXC1, and HDAC1, thereby blocking the terminal differentiation of myeloid cells and leading to AML development (Figure 6) (Simeoni and Somervaille, 2021). TLE3 may suppress Wnt signaling as TLE1/4 does, but this activity could be attenuated in AML cells through mechanisms similar to those in solid tumor cells, which will be described in the next section.

In T-ALL, the survival of cancer cells depends on continuous activation of the Notch pathway (Hales et al., 2014). Notch likely inhibits the transcription of PTEN through the HES1 and TLE complex and the recruitment of HDACs (Wong et al., 2012; Pappas et al., 2021; Zhang et al., 2022). PTEN inhibition consequently enhances PI3K/Akt signaling activation and promotes the survival of T-ALL cells (Wong et al., 2012; Pappas et al., 2021; Zhang et al., 2022). In addition, Notch/HES1 suppresses the expression of CYLD, a negative regulator of IKK activity, leading to sustained NF-κB activation and increased cell survival (Figure 6) (D'Altri et al., 2011); however, whether it depends on the HES1/TLE complex has yet to be verified. Other effects downstream of Notch activation may also contribute to T-ALL development, such as decreased E47 and increased oncogene MYC expression (Figure 6) (Sanchez-Martin and Ferrando, 2017). While MYC seems to be upregulated as a direct Notch target gene, the mechanisms underlying E47 suppression are not fully understood (Koch and Radtke, 2011). TLE1 might inhibit Notch-induced MYC gene transcription, but TLX1, a highly expressed TF in T-ALL, can form a complex with TLE1, thus derepressing MYC gene expression (Riz et al., 2010). Therefore, TLE1 may have tumor suppressive roles in T-ALL development, consistent with the observation that low TLE1 expression is associated with poorer survival in T-ALL patients (Brassesco et al., 2018; Aref et al., 2021). Targeting Notch produced a significant anticancer effect in T-ALL with prominent Notch activation (Koch and Radtke, 2011); however, T-ALL with PTEN mutations did not respond to the treatment (Palomero et al., 2007; Hales et al., 2014), demonstrating that Notch-induced PTEN inhibition is essential for T-ALL cell survival.

### Mechanisms by which TLEs suppress solid tumors

**FIGURE 7:**
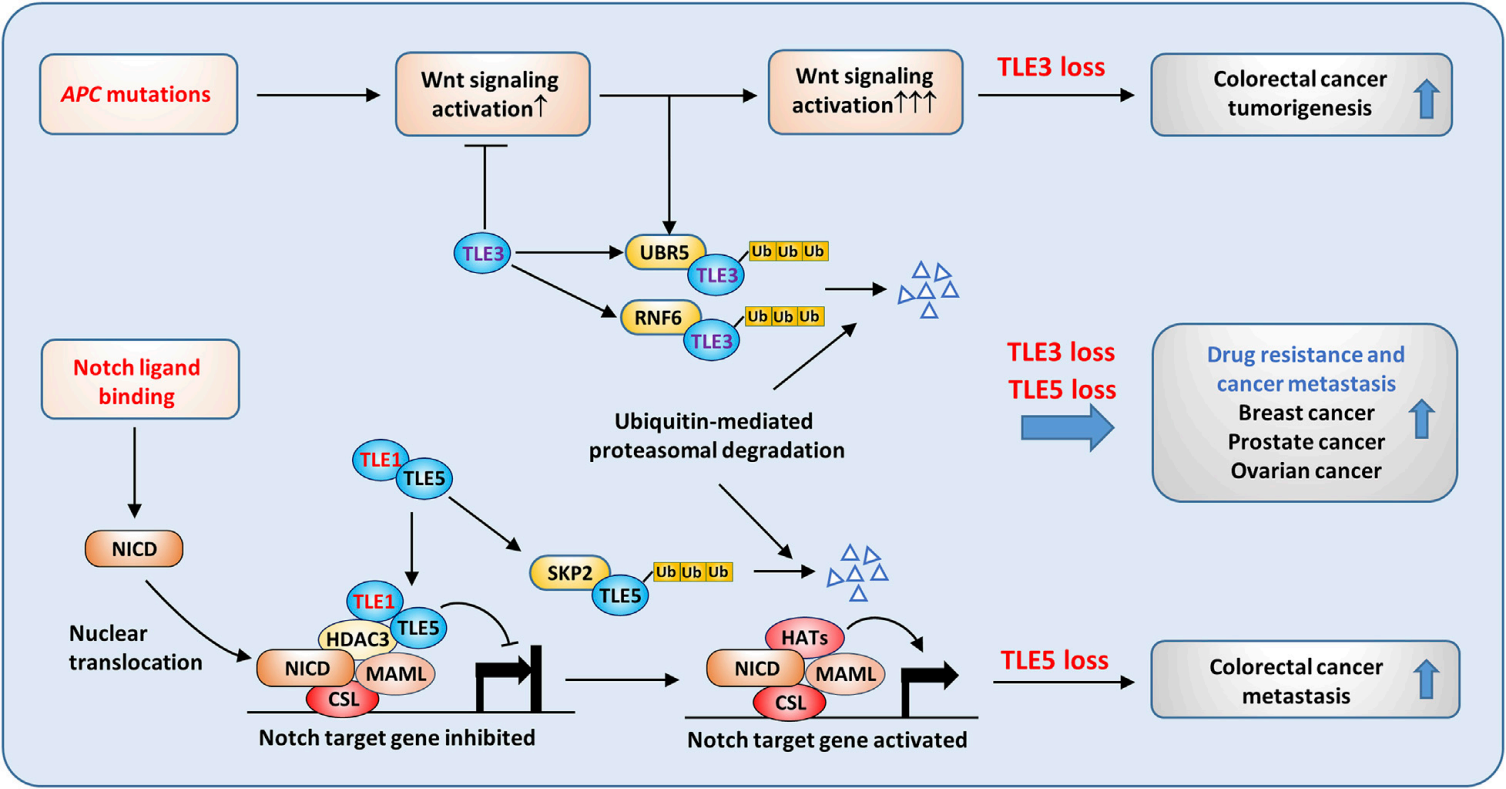
Tumor suppressive roles of TLEs in solid tumors. Aberrant Wnt signaling activation caused by APC mutation is common in colorectal cancer. TLE3 suppresses Wnt activation in normal epithelial cells, but in cancer cells, ubiquitin ligases, such as UBR5 and RNF6, are upregulated and are responsible for TLE3 ubiquitination and degradation. As a result, loss of TLE3 promotes colorectal tumorigenesis by enhancing Wnt activation. TLE5 suppresses colorectal cancer metastasis by inhibiting Notch signaling. SKP2-mediated TLE5 ubiquitination and degradation lead to Notch activation and tumor metastasis. Moreover, loss of TLE3 and TLE5 is frequently associated with drug resistance and metastasis in breast, prostate, and ovarian cancers.

TLEs may exert tumor suppressive activity in solid tumors and are regulated by distinctive mechanisms. As expected, the inhibitory effect of TLEs on Wnt target gene expression contributes to the antitumoral activities of TLEs in certain types of solid tumors. For example, UBR5 is a highly conserved HECT-domain E3 ubiquitin ligase that is frequently overexpressed in several cancer types, including colon (Ji et al., 2017), ovarian (Song et al., 2020), pancreatic (Chen et al., 2021), and breast (Liao et al., 2017) cancers. Flack et al. showed that UBR5 promotes Wnt activation by inducing the ubiquitylation and subsequent degradation of TLE3 (Flack et al., 2017). In addition, RNF6 is a RING-type E3 ubiquitin-protein ligase, and its genetic amplification and overexpression are prevalent in colorectal cancer (CRC). High RNF6 expression was shown to promote CRC cell proliferation and metastasis, and the effect was related to RNF6-mediated TLE3 ubiquitination followed by enhanced Wnt signaling (Liu et al., 2018). Moreover, Hanson et al. reported that XIAP, another E3 ligase, induces monoubiquitylation of TLE3 and blocks the interaction of TLE3 and TCF4, thus promoting Wnt signaling activation in CRC cells (Hanson et al., 2012). These results demonstrate that TLE3 is critical in regulating Wnt signaling in tumorigenesis (Figure 7). As previously mentioned, TLE3 expression is linked to increased sensitivity to chemotherapy and targeted therapy in several cancer types. In prostate cancer, loss of TLE3 is associated with resistance to AR inhibitors. Further study revealed that the expression of the glucocorticoid receptor (GR) gene was depressed by AR and TLE3 in prostate cancer cells. In the absence of TLE3, further inhibition of AR led to the derepression of GR expression, which confers resistance to AR-targeted therapy in prostate cancer (Palit et al., 2019).

TLE5 also plays a tumor suppressive role in clonal cancer metastasis. TLE5 overexpression inhibited Notch signaling activation through recruitment of TLE1 and HDAC3, converting active Notch transcriptional complexes into repressive complexes, which reduced the expression of Notch target genes such as HES1 in clone cancer cells (Figure 7) (Sonoshita et al., 2011). Consistently, knocking down TLE5 enhanced tumor invasiveness by activating Notch signaling. Intriguingly, TLE1 alone could not suppress Notch signaling in colon cancer cells, but it could potentiate the suppressive activity of TLE5 by forming distinct nuclear foci containing TLE1, TLE5, and HDAC3 (Figure 7). Similarly, TLE5 suppressed prostate cancer metastasis by inhibiting AR and Notch signaling, and loss of TLE5 promoted tumor invasion and metastasis by increasing Snail and MMP9 expression (Okada et al., 2017).

The ubiquitin‒proteasome degradation system seems to play a critical role in regulating TLE3 levels; however, the mechanisms by which TLE5 is suppressed in metastatic colon and prostate cancers are not fully understood. Kakizaki et al. found that TLE5 gene expression was activated by the Yin-yang transcription factor YY2, which is frequently downregulated in metastatic colon cancer (Kakizaki et al., 2016). Recently, Wang et al. found that TLE5 was phosphorylated by CK1δ/ε, which promoted SKP2-mediated ubiquitination and degradation of TLE5 (Wang et al., 2021a). CK1δ and CK1ε are overexpressed in colon cancer and are inversely correlated with low TLE5 expression. Consistently, treatment with a CK1 inhibitor suppressed tumor growth by stabilizing TLE5 (Wang et al., 2021a). These data suggest that both transcriptional and posttranslational mechanisms are involved in regulating TLE5 levels in cancer.

## The roles of TLEs in immune cell differentiation and immune regulation

### TLEs regulate inflammatory gene expression in macrophages

Macrophages are crucial mediators of inflammatory responses. The onset, magnitude, and resolution of inflammatory responses are orchestrated by macrophages whose activities are controlled by an integrated pathway network, in which NF-κB pathway activation downstream of toll-like receptors (TLRs) plays a central role (Dorrington and Fraser, 2019; Zinatizadeh et al., 2021). In addition, macrophages can sense different environmental stimuli and differentiate into functionally different subgroups (Porta et al., 2015; Locati et al., 2020). For example, stimulation with the TLR4 agonists lipopolysaccharide (LPS) and IFN-γ induces the differentiation of M1-type macrophages, which secrete proinflammatory cytokines and can kill pathogens directly through phagocytosis or indirectly by activating T-cell-mediated immune responses. In contrast, IL-4 and IL-13 promote the differentiation of M2-type macrophages, which mainly exert anti-inflammatory functions by secreting immunosuppressive cytokines (Murray et al., 2014).

Recent studies revealed that TLEs play critical roles in inflammation by regulating macrophage activation and differentiation. For instance, in M2 macrophages derived from the THP1 human monocyte line, TLE1 expression was significantly upregulated, inhibiting the transcription of proinflammatory genes in M2 macrophages (De Paoli et al., 2016). Tle1^−/−^ mice exhibited an inflammatory phenotype; that is, the levels of proinflammatory cytokines, including IL-1β and IL-6, were systemically elevated (Ramasamy et al., 2016). Macrophages isolated from the Tle1^−/−^ mice produced more inflammatory cytokines upon LPS stimulation *in vitro* (Ramasamy et al., 2016). In addition, Zhang et al. found that the interaction between TLE4 and HES1 attenuated the expression of proinflammatory cytokines, including IL-6 and IL-12, in murine macrophages (Zhang et al., 2019).

TLR and Notch signaling pathway components in macrophages engage in a complex interaction (Palaga et al., 2008; Foldi et al., 2010; Zhang et al., 2012; Gamrekelashvili et al., 2020). TLEs may suppress the expression of inflammatory cytokines in macrophages by regulating the crosstalk between the TLR and Notch pathways. Many studies have suggested that TLR pathway activation promotes Notch expression. For instance, LPS stimulation increased the expression of Notch receptors and HES1 in macrophages, and in turn, Notch activation enhanced the phosphorylation of NF-κB (Foldi et al., 2010; Lopez-Lopez et al., 2020). Conversely, Notch activation induced by NICD overexpression promoted the nuclear translocation of NF-κB and thus enhanced the expression of NF-κB target genes in macrophages (Foldi et al., 2010). In addition, Notch1 activation enhanced basal and LPS-induced NF-κB activation, augmenting the expression of proinflammatory cytokines (Monsalve et al., 2009), whereas inhibiting Notch by depleting RBP-J or by treatment with a γ-secretase inhibitor (GSI) suppressed proinflammatory cytokine expression in macrophages (Hu et al., 2008; Palaga et al., 2008). These results suggest positive feedback between Notch and NF-κB signaling in LPS-stimulated macrophages, leading to increased inflammatory cytokine production.

Zhang et al. found that LPS stimulation enhanced Notch1, Notch2 and HES1 expression in macrophages (Zhang et al., 2012). However, in contrast to the observations of Monsalve’s group (Monsalve et al., 2009), Zhang et al. found that Notch activation induced by NICD overexpression inhibited the LPS-induced expression of TNF-α and IL-6 by suppressing ERK activity (Zhang et al., 2012). Notably, LPS stimulation enhanced the expression of HES1 in macrophages, and depleting HES1 in macrophages enhanced cytokine production upon LPS stimulation, suggesting that HES1 exerts a negative feedback effect on TLR pathway activation (Hu et al., 2008). Therefore, under certain circumstances, Notch activation antagonizes TLR activation in macrophages. Interestingly, IFN-γ attenuated the HES1 gene expression upregulation induced by LPS (Hu et al., 2008), suggesting that IFN-γ may augment LPS-induced cytokine expression by blocking HES1-mediated negative feedback. Therefore, with regard to the complicated interaction between the Notch and TLR signaling pathways in macrophages, some observations seem inconsistent, likely due to different experimental conditions or the sources and states of the macrophages used. Another explanation suggests that the inhibitory effects of Notch activation on the TLR/NF-κB pathway depend on the availability of HES1 and TLE1. As mentioned earlier, HES1 and TLE1 form a stable complex that regulates the Notch pathway. In agreement with that, Larabee et al. found that cAMP, which exerts an immunosuppressive effect, increased RBP-J and TLE1 expression and inhibited the expression of inflammatory cytokines in macrophages by activating the Notch pathway (Larabee et al., 2013).

**FIGURE 8:**
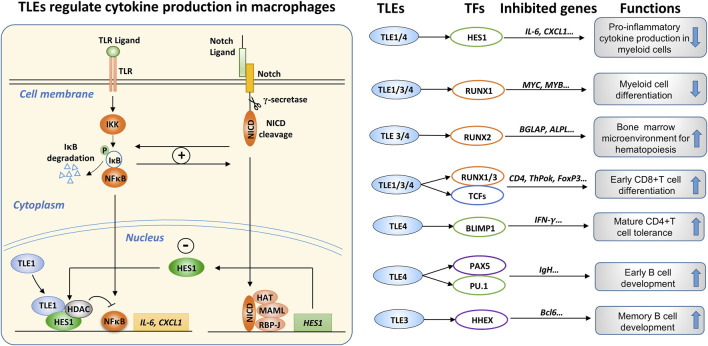
The roles of TLEs in immune regulation. (Left): TLEs are involved in the interconnection between the TLR and Notch pathways in macrophages. The engagement of TLR ligands activates IKKα/β kinase, inducing the phosphorylation and subsequent degradation of IκB, which sequesters NF-κB transcription factors (TFs) in the cytoplasm. Consequently, the released NF-κB TFs, mainly p65 and p50, are translocated from the cytoplasm to the nucleus, where they stimulate the expression of proinflammatory cytokines, such as IL-6 and CXCL1. Notch activation induces the expression of target genes, including HES1. The TLR and Notch pathways can regulate each other through a positive feedback loop; however, Notch activation induces the expression of HES1, which can form a transcriptional suppressive complex with TLE1/4 and HDACs, attenuating the expression of inflammatory cytokines. Therefore, TLE1/4, and possibly other TLE family members, are involved in the intricate interconnection between the TLR and Notch pathways in macrophages. (Right): Summary of the roles of TLEs in immune regulation, depicting TLE members, interacting TFs, suppressed target genes, and effects, including cytokine production in myeloid cells (Ramasamy et al., 2016), myeloid lineage differentiation (Simeoni and Somervaille, 2021), hematopoiesis (Wheat et al., 2014), early T cell differentiation (Xing et al., 2018), T cell tolerance (Bandyopadhyay et al., 2014), and early and memory B cell development (Milili et al., 2002; Laidlaw et al., 2020).

Notably, the expression of inflammatory cytokines in Tle1^−/−^ macrophages increased significantly after LPS stimulation (Ramasamy et al., 2016). Further analysis revealed that HES1 protein levels in Tle1^−/−^ macrophages were significantly decreased regardless of LPS stimulation but the HES1 mRNA level was increased, suggesting that the decrease in the HES1 protein level was not due to reduced gene transcription (Ramasamy et al., 2016). We speculate that TLE1 may inhibit degradation of HES1 protein by forming a complex with HES1; then, the TLE1/HES1 complex may be recruited to cytokine genes to suppress their expression. Recently, Hes1^−/−^ murine macrophages were shown to produce more type I interferon and inflammatory factors, including IL-6, IL-12, and CXCL1, although the role played by TLEs was not investigated (Zhang et al., 2019). Based on these findings, we can infer that TLE1, and likely TLE4, forms an inhibitory complex with HES1, which serves as an essential feedback mechanism to prevent macrophage overactivation, as depicted in Figure 8.

As mentioned above, Tle4^−/−^ mice suffered early postnatal death due to several hematopoietic and skeletal defects at approximately 4 weeks (Wheat et al., 2014). Zhang et al. independently generated Tle4^−/−^ mice and found that some of the mice died early from developmental defects in the skeletal system. Nevertheless, they found that hematopoiesis in the surviving Tle4^−/−^ mice was essentially normal (Zhang et al., 2019). Because of the very similar knockout strategies (exon two deletion) used to generate the Tle4^−/−^ mice (Wheat et al., 2014; Zhang et al., 2019), the cause of the discrepancy is unclear. Zhang et al. found that after LPS stimulation, the levels of TLE1 and TLE2 in mouse bone marrow-derived macrophages (BMDMs) changed negligibly, but TLE3 and TLE4 levels increased significantly in an NF-κB and JNK pathway-dependent manner (Zhang et al., 2019). Using immortalized mouse BMDM cell lines, they also found that depleting TLE4 increased IL-6 and IL-12 production by macrophages but depleting TLE1, TLE2, and TLE3 simultaneously produced little effect, which seems inconsistent with the increased cytokine production in Tle1^−/−^ primary macrophages (Ramasamy et al., 2016), potentially because of the different cell sources used.

### Expression and function of TLEs in other immune cell types

Interestingly, the surviving Tle1^−/−^ and Tle4^−/−^ mice showed abnormal hematopoiesis, but their phenotypes were distinct. The interaction between TLE1 and HHEX was reported to be critical for the early differentiation of hematopoietic stem cells (Swingler et al., 2004); however, in Tle1^−/−^ mice, only the myeloid lineage showed enhanced differentiation, and the phenotype was not acquired through intrinsic defects in hematopoietic cells (Ramasamy et al., 2016). In contrast, TLE4 deficiency diminished the hematopoietic stem cell pool and caused leukocytopenia, especially affecting B cells (Wheat et al., 2014). This finding can be partially explained by the fact that TLE4 is expressed at a higher level than TLE1 in B-cell progenitors (Milili et al., 2002). PAX5 is a critical TF in B-cell development, and the inhibitory effect of PAX5 on target gene expression in developing B cells depends on the interaction of TLE4, PAX5, and PU.1 (Eberhard et al., 2000; Milili et al., 2002; Linderson et al., 2004). Notably, the hematopoietic abnormalities in Tle4^−/−^ mice were more extensive than those in Pax5-knockout mice (Nutt et al., 2001), suggesting that other TLE4-interacting factors may be involved. Moreover, TLE3 and TLE4 regulate osteoblast differentiation of bone marrow stromal cells and bone development by binding RUNX2 (Kokabu et al., 2013; Wheat et al., 2014; Shin et al., 2021a). Since hematopoiesis and normal bone development are intimately connected, TLE3/4 may regulate hematopoiesis *via* intrinsic and extrinsic mechanisms.

As mentioned earlier, both TLE1 and TLE4 can bind HES1 and mediate Notch signaling effects (Grbavec and Stifani, 1996; Jennings et al., 2006; Dayyani et al., 2008; Zhang et al., 2019), and Notch activation inhibits myeloid cells while promoting T-cell development (Radtke et al., 2004; de Pooter et al., 2006; Cheng et al., 2014; De Decker et al., 2021). Therefore, the enhanced myeloid differentiation in Tle1^−/−^ mice might be associated with suppressed Notch effects (Ramasamy et al., 2016). In Tle4^−/−^ mice, the hematopoietic abnormality was more extensive, likely due to broader expression of TLE4 (Wheat et al., 2014). Global deletion of Tle3 causes embryonic death, and the role of TLE3 in immune cell differentiation and function remains largely unknown. Laidlaw et al. found that TLE3 binds to HHEX and promotes germinal center B cell differentiation into memory B cells by inhibiting Bcl6 expression (Laidlaw et al., 2020).

Progenitor T cells in the thymus undergo a double-negative (DN, CD4^−^CD8^−^) stage and a double-positive (DP, CD4^+^CD8^+^) stage and finally differentiate into either CD4^+^ or CD8^+^ single-positive mature T cells. Xing et al. found that T cells mainly expressed TLE3 and TLE4, with TLE3 expressed at a higher level, while TLE1 and TLE2 expression was very low (Xing et al., 2018). Since global Tle3 knockout resulted in mouse embryonic death (Laing et al., 2015), this group prepared mice with Tle3 conditional knockout in hematopoietic cells and found that the number of CD8^+^ single-positive cells decreased while the number of CD4^+^ single-positive cells increased in the thymocyte population, suggesting that TLE3 was very important for the development of CD8^+^ T cells (Xing et al., 2018). To address the functional redundancy among TLE family members, the authors prepared Tle1-, Tle3-, and Tle4-triple-knockout (TKO) mice using Cd4-cre mice to delete the three genes at the DP stage of T-cell development. The results indicated that the thymus and peripheral blood of the Cd4-cre-TKO mice generated a negligible number of CD8^+^ T cells but an increased number of CD4^+^ T cells, demonstrating that TLEs are critical for differentiation of the CD8^+^ T lineage (Xing et al., 2018). TLE3 seems to play a dominant role in T-cell development (Xing et al., 2018), which explains why no significant abnormalities were observed in the T cells of the Tle1 and Tle4 single-gene-knockout mice (Wheat et al., 2014; Ramasamy et al., 2016).

RUNX family proteins (RUNX1-3) are critical for the development of various immune cells (Seo and Taniuchi, 2020; Shin et al., 2021b; Korinfskaya et al., 2021). The loss of CD8^+^ T cells in the Tle1-, Tle3-, and Tle4-TKO mice led to a phenotype similar to that of Runx1/Runx3-double-knockout mice (Xing et al., 2018), which is consistent with the observation that RUNX1/3 suppresses the differentiation of CD4^+^ T cells (Telfer et al., 2004; Yarmus et al., 2006). Further analysis showed that TLEs inhibited the expression of genes related to CD4^+^ T-cell differentiation by interacting with RUNX1/3, thus promoting the differentiation of early T-cell progenitors into the CD8^+^ T-cell lineage (Xing et al., 2018). RUNX1 and RUNX3 play essential roles in the antigen-dependent immune response of T cells (Korinfskaya et al., 2021), but how TLEs affect the activity of mature T cells has not been investigated. RUNX2 is extremely important in osteogenesis during bone development, and its critical roles in other biological processes are emerging. RUNX2 is overexpressed in various malignancies, including high-risk T-ALL, B-cell non-Hodgkin’s lymphoma, and multiple myeloma (Zhang et al., 2020). In addition, RUNX2 regulates the development and migration of plasmacytoid dendritic cells (Sawai et al., 2013; Chopin et al., 2016). Omatsu et al. reported that RUNX1 and RUNX2 were required for the maintenance of hematopoietic stem cells in bone marrow (Omatsu et al., 2022). RUNX2 is also required for the generation and tissue residency of human NK cells (Wahlen et al., 2022).

Further studies are warranted to dissect the roles of TLE/RUNX interactions in cancer and immune regulation. In Figure 8 (right), we summarize the interaction of TLEs and TFs, the suppressed genes, and the observed effects on immune cell development and function. In conclusion, multiple TLE isoforms may have overlapping functions in immune regulation. Genetic depletion of individual TLE isoforms usually causes significant defects in animal development, making it challenging to study the overall function of specific TLE isoforms in the immune system. In the future, conditional knockout mice will help delineate the function of TLE isoforms in immune regulation (Wang et al., 2004; Yu et al., 2014; Okada et al., 2017; Feng et al., 2020).

## Perspectives on regulating TLEs for cancer therapy

### Targeted therapy

TLEs regulate several signaling pathways involved in cancer cell survival and drug resistance. Therefore, it is speculated that modifying TLEs could improve the outcome of cancer therapies. Taking EGFR targeted therapy as an example, it is known that EGFR pathway activation induces Gro/TLE protein phosphorylation and reduces their suppression activity and that Gro/TLEs mediate the mutual antagonism between EGFR and Notch signaling pathways (Hasson et al., 2005; Hasson and Paroush, 2006). EGFR gene amplification and overexpression are frequent in several cancer types, including lung adenocarcinomas, colon carcinomas, and pancreatic adenocarcinomas (Sigismund et al., 2018). In lung adenocarcinoma, activating EGFR gene mutations have been identified as the main oncogenic factors, especially in Asian countries (Zhou and Zhou, 2018). Targeted therapy with EGFR inhibitors has shown a significant therapeutic effect in patients with EGFR-mutant lung cancer (Bartholomew et al., 2017). However, most patients develop resistance to EGFR inhibitors, which significantly limits the long-term benefits of this targeted therapy (Bartholomew et al., 2017). Codony-Servat et al. found that EGFR inhibition activated Notch signaling and thus induced the outgrowth of tumor stem cells and that the efficacy of EGFR-targeted therapy diminished in tumors with high HES1 expression (Codony-Servat et al., 2019). Protein kinase 2 (CK2) is involved in HES1-dependent TLE hyperphosphorylation, which is critical for TLE suppressive activity (Nuthall et al., 2004). Notably, CK2 is frequently overexpressed in many cancers and is a valuable pharmacological target (Salvi et al., 2021). More investigation is needed to investigate whether targeting CK2 can inhibit TLE hyperphosphorylation in the setting of EGFR-targeted therapy.

In addition, EMT is involved in acquired resistance to EGFR-targeted therapy (Tulchinsky et al., 2019; Zhu et al., 2019), and Notch activation is closely related to EMT transformation (Xie et al., 2012; Shao et al., 2015). Wnt pathway activation also promotes EMT by upregulating several EMT-activating TFs, including Slug, ZEB1, and VIM (Togashi et al., 2015; Basu et al., 2018). In addition, Rajeswara et al. found that EGFR inhibition induced high expression of Notch3 and *ß*-catenin (Arasada et al., 2018). Together, these studies suggest that resistance to EGFR inhibitors is related to the feedback activation of the Notch and Wnt pathways. As mentioned earlier, TLEs regulate both the Notch and Wnt/β-catenin pathways by interacting with HES1 and TCFs, respectively; therefore, it will be interesting to examine the roles of TLEs in resistance to EGFR-targeted therapy in the future.

### Immunotherapy

Macrophages are abundant in tumor tissues and are often collectively referred to as tumor-associated macrophages (TAMs). Due to an immunosuppressive tumor microenvironment, TAMs more often differentiate into M2-type macrophages, which are believed to promote tumor progression (Shu and Cheng, 2020; Bart et al., 2021). NF-κB activation is essential for differentiation of TAMs into M1 macrophages with antitumor activity (Taniguchi and Karin, 2018; Dorrington and Fraser, 2019). In early lung cancer, NF-κB plays an essential role in immune surveillance (Hopewell et al., 2013), and the activation of M1 macrophages is critical to inhibiting the progression of this disease (Singhal et al., 2019). Moreover, the activation of NF-κB in T cells is necessary for their antitumor function (Eluard et al., 2020). As mentioned above, TLE1 may inhibit NF-κB activation (Tetsuka et al., 2000; Ghosh et al., 2007; Ramasamy et al., 2016; Chen et al., 2020) and suppress macrophage proinflammatory activity, which is closely related to M1-type macrophage differentiation (De Paoli et al., 2016; Ramasamy et al., 2016; Zhang et al., 2019). TLE1 is frequently upregulated and may function as an oncogene in certain cancer types, such as NSCLC, invasive breast cancer, and GMB (Allen et al., 2006; Brunquell et al., 2012; Holmes et al., 2012; Verginelli et al., 2013; Yao et al., 2014a; Yao et al., 2016; Lee et al., 2017). Hence, targeting TLE1 in these tumors, especially at an early stage, may directly suppress the oncogenic activity of TLE1 and promote TAM anticancer activities.

Other types of immune cells, including T cells and B cells, are also essential components of the tumor microenvironment and significantly impact tumor progression. TLEs significantly impact the development of T cells and B cells, but whether they affect the function of mature cells in tumors is largely unknown. A recent study showed that depleting TLE4 expression significantly enhanced the antitumor activity of chimeric antigen receptor T (CAR-T) cells (Wang et al., 2021b). TLEs are expressed in other types of immune cells, such as natural killer (NK) cells and dendritic cells, and therefore, more study is needed to investigate the roles played by TLEs in these immune cells, ideally in the context of the tumor immune microenvironment, to determine whether targeting TLEs can be developed into a novel anticancer therapy.

## Conclusion and future directions

No TLE-specific inhibitors are currently available; however, some strategies might help modulate TLE activity in future studies. For example, diverse posttranslational modifications of TLEs, including phosphorylation (Choi et al., 2005; Ciarapica et al., 2014), ubiquitination (Nuthall et al., 2004; Flack et al., 2017), and SUMOylation (Ahn et al., 2009; Lee et al., 2012), can negatively or positively influence TLE function. As mentioned above, ERK-induced phosphorylation weakens Gro/TLE suppressive activity (Hasson et al., 2005; Zahavi et al., 2017). Thus, it is possible to reactivate TLEs by blocking ERK activation using inhibitors of the RTK/MAPK pathway. This strategy may directly inhibit oncogenic MAPK pathway activation and indirectly modify Notch and Wnt pathway activity, thereby exerting an enhanced anticancer effect in some cancer types. In addition, it appears that the modification of TLEs by ubiquitin ligases plays a role in several cancers. Various ubiquitin ligases, such as XIAP, UBR5, RNF6, and SKP2, are responsible for the reduced TLE levels in some tumors. Whether targeting relevant ubiquitin ligases could improve the outcome of cancer therapy needs further investigation.

In conclusion, we now know that individual TLE members are dynamically expressed in different cell contexts and that their levels are extensively controlled through multiple mechanisms. In addition, TLEs are critical modulators in multiple signaling pathways rather than permissive corepressors and are actively involved in tumorigenesis and immune regulation. TLEs may have oncogenic or antitumor activities in cancers of different tissue origins, and based on their intricate relationship with tumors and immunity, TLEs can significantly impact the tumor immune microenvironment (TIM). Therefore, more studies are needed to better understand the regulation and function of TLEs in tumorigenesis and immune system activity, which is a prerequisite for successfully inhibiting TLEs in certain diseases or reactivating them in others.
